# Supplementary material for: Accounting for diverse evolutionary forces reveals mosaic patterns of selection on human preterm birth loci
Source: Nat Commun. 2020 Jul 24;11:3731. doi: 10.1038/s41467-020-17258-6 (PMC7382462; doi:10.1038/s41467-020-17258-6)
Supplement: Supplementary file 1 — Supplementary Information [file 41467_2020_17258_MOESM1_ESM.pdf]

Supplementary Information

Accounting for diverse evolutionary forces reveals mosaic patterns of selection on human preterm birth loci

LaBella, Abraham et al.

Table of Contents:

Supplementary Table:
1. Number of sPTB regions with evolutionary signatures

Supplementary Figures:
1. Match quality of sPTB regions across control regions
2. Matched and unmatched distributions for diverse evolutionary measures
3. Sensitivity analysis of diverse evolutionary forces acting on sPTB-associated genomic regions

Supplementary Table 1: Number of sPTB regions with evolutionary signatures. Number of sPTB regions out of 215 that have evidence for evolutionary signatures (top 5th percentile) when compared to 1,000 iteration of randomly selected regions (n=5,0000 per PTB region) for each evolutionary measure.

Evolutionary Measure	Mean	Standard Deviation
ARGweaver-TMRCA	39.5	1
Beta Score	23.9	0.2
F_{ST} Eas-Afr	30.2	0.5
F_{ST} Eur-Afr	36	0.3
F_{ST} Eas-Eas	32.5	0.7
GERP	23.4	0.8
iES Sabeti Eur	33	0.2
LINSIGHT	33.3	0.8
PhastCons	19	0.1
PhyloP	19.5	0.8
XP-EHH Afr-Eas	20	0.1
XP-EHH Afr-Eur	16.5	0.7
XP-EHH Eas-Eas	15.3	0.7

Supplementary Figure 1: Match quality of sPTB regions across control regions. For each 215 sPTB-associated genomic regions, we generated control regions matched on minor allele frequency and linkage disequilibrium structure. The match quality per genomic region, defined as the fraction of sPTB variants with a matched variant averaged across all control regions, is ≥ 99.6% for all sPTB-associated genomic regions (Figure 1B).

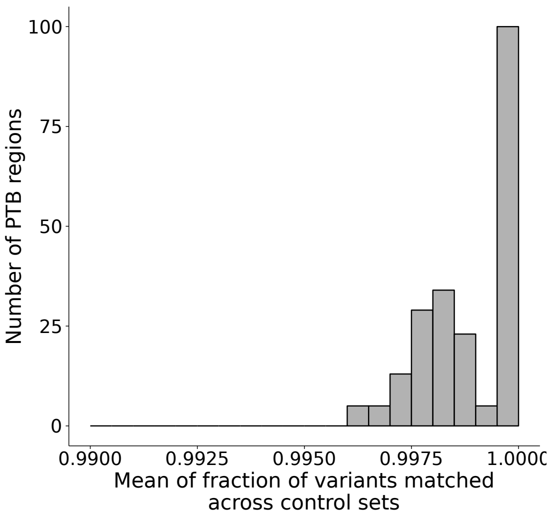

Supplementary Figure 2: Matched and unmatched distributions for diverse evolutionary measures.

Each figure below includes sPTB-associated genomic regions labeled by the lead variant (x-axis) that was statistically significant using either a genome wide background (blue dot), a matched background (red dot), or both (black dot) for the median value for a specific evolutionary measure (y-axis). Genome wide background distribution ('Unmatched Bkg, x-axis) is the filled violin plot; all unfilled violin plots are the MAF and LD matched distributions labeled by the lead variant for that sPTB genomic region. Dotted horizontal lines are genome wide unmatched thresholds for one or two-sided empirical p-value of 0.05 calculated from the genome-wide background distribution. Gold horizontal lines for each violin plot represents the threshold for empirical p-values of 0.05 derived from the matched distributions. Allele age (TMRCA from ARGweaver), PhyloP, and alignment block age are bi-directional measures, therefore, we calculated two-tailed p-values; all other evolutionary measures used one-tailed p-values. The evolutionary measures tested with sPTB regions include (a) ARGweaver-TMRCA, (b) Beta Score, (c)F_{ST} Eas-Afr, (d) F_{ST} Eur-Afr, (e) F_{ST} Eur-Eas (f) GERP, (g) iES, (h) LINSIGHT, (i) PhastCons, (j) PhyloP, (k) Syntentic age, (l) XP-EHH Afr-Eas, (m) XP-EHH Afr-EUR, (n) XP-EHH Eas-Eur.

a)

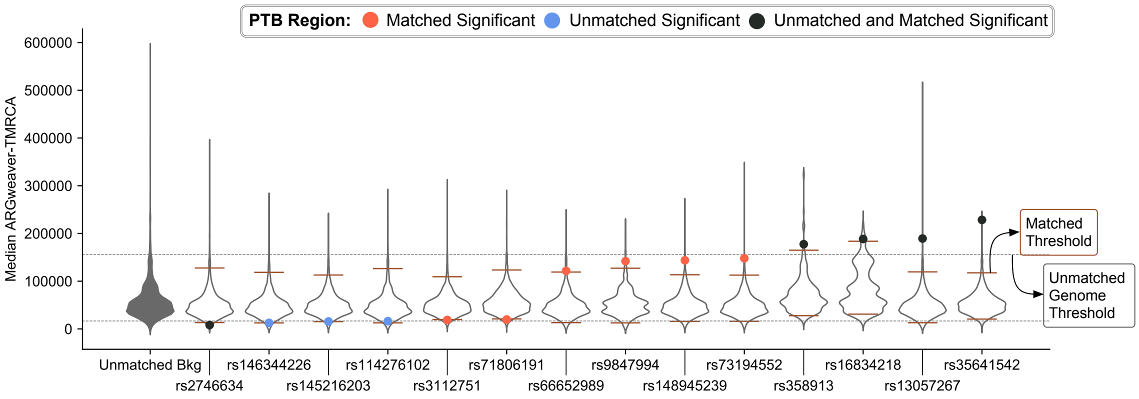

b)

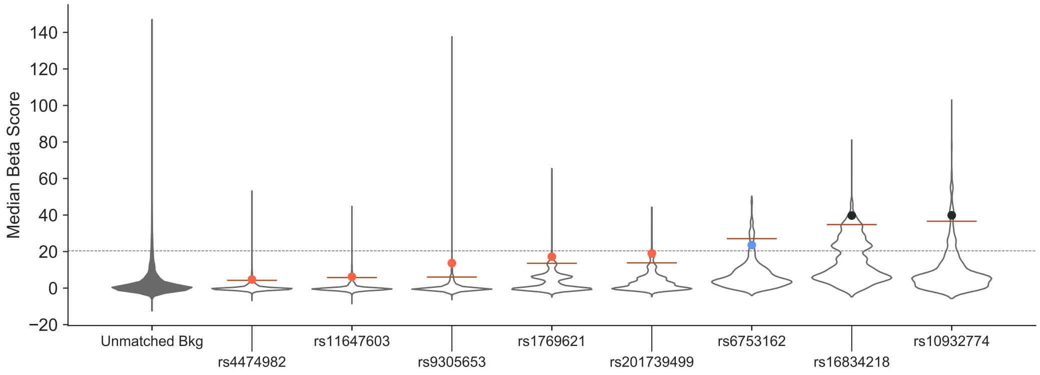

c)

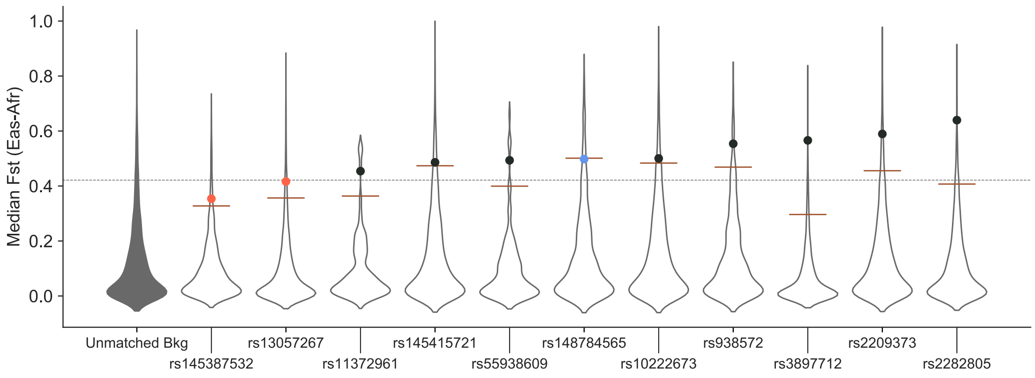
Median Fst (Eas-Afr)
1.0
0.8
0.6
0.4
0.2
0.0
Unmatched Bkg
rs145387532
rs13057267
rs11372961
rs145415721
rs55938609
rs148784565
rs10222673
rs938572
rs3897712
rs2209373
rs2282805

d)

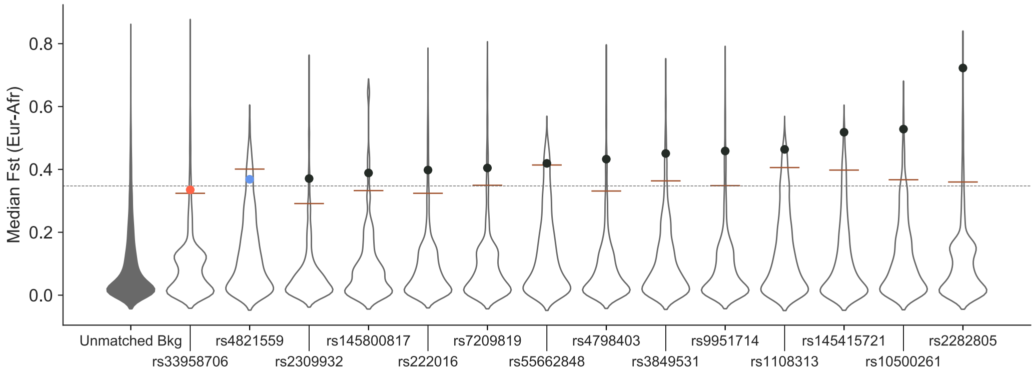
Median Fst (Eur-Afr)
0.8
0.6
0.4
0.2
0.0
Unmatched Bkg
rs33958706
rs4821559
rs2309932
rs145800817
rs222016
rs7209819
rs55662848
rs4798403
rs3849531
rs9951714
rs1108313
rs145415721
rs10500261
rs2282805

e)

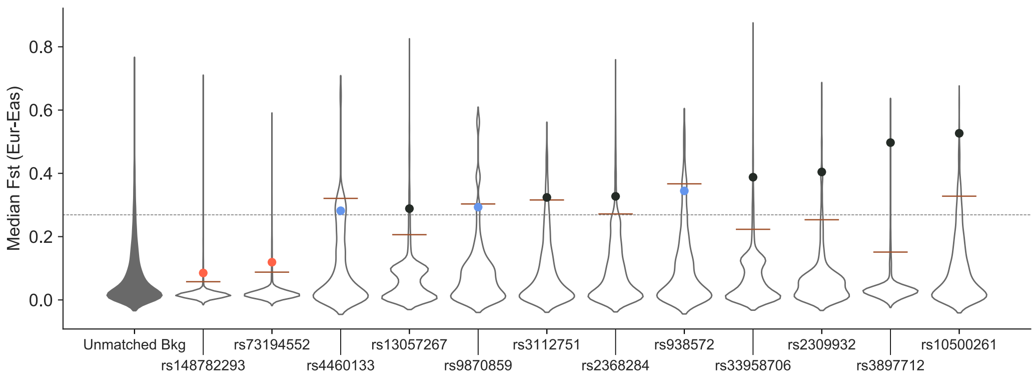
Median Fst (Eur-Eas)
0.8
0.6
0.4
0.2
0.0
Unmatched Bkg
rs148782293
rs73194552
rs4460133
rs13057267
rs9870859
rs3112751
rs2368284
rs938572
rs33958706
rs2309932
rs3897712
rs10500261

f)

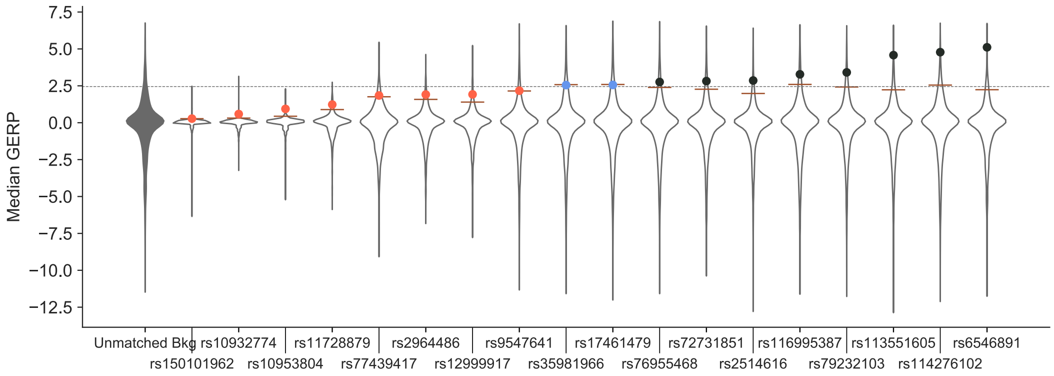
Median GERP
7.5
5.0
2.5
0.0
−2.5
−5.0
−7.5
−10.0
−12.5
Unmatched Bkg
rs150101962
rs10932774
rs10953804
rs11728879
rs77439417
rs2964486
rs12999917
rs9547641
rs35981966
rs17461479
rs76955468
rs72731851
rs2514616
rs116995387
rs79232103
rs113551605
rs114276102
rs6546891

g)

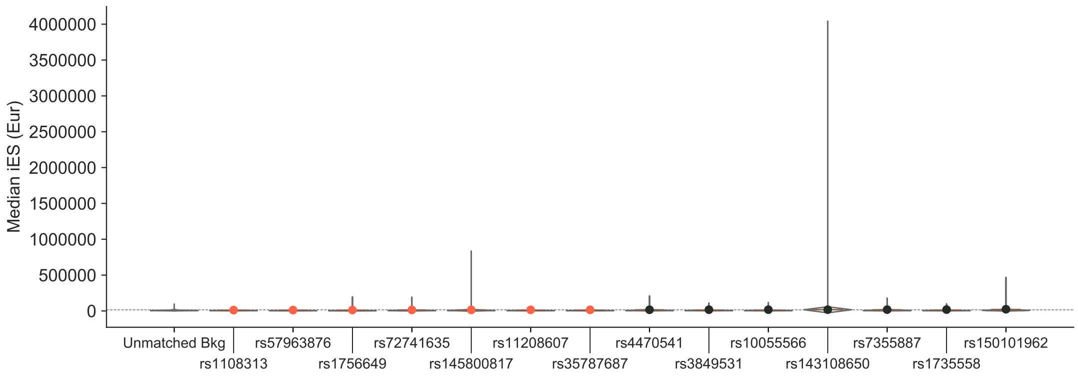
Median iES (Eur)
4000000
3500000
3000000
2500000
2000000
1500000
1000000
500000
0
Unmatched Bkg
rs1108313
rs57963876
rs1756649
rs72741635
rs145800817
rs11208607
rs35787687
rs4470541
rs3849531
rs10055566
rs143108650
rs7355887
rs1735558
rs150101962

h)

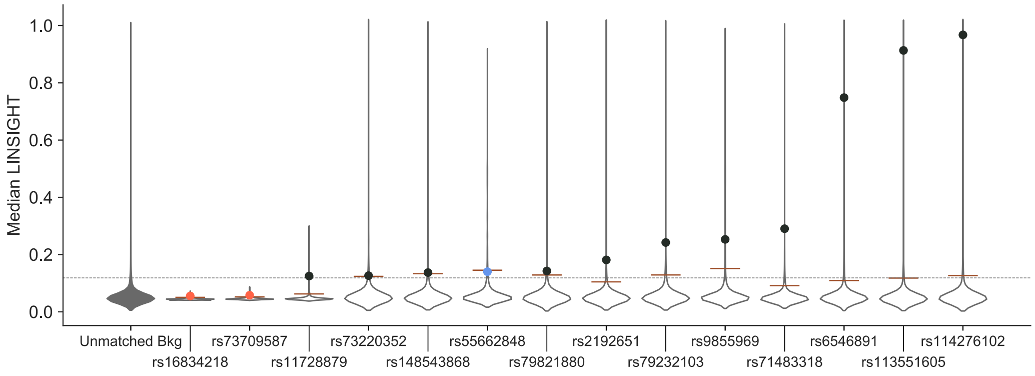
Median LINSIGHT
1.0
0.8
0.6
0.4
0.2
0.0
Unmatched Bkg
rs16834218
rs73709587
rs11728879
rs73220352
rs148543868
rs55662848
rs79821880
rs2192651
rs79232103
rs9855969
rs71483318
rs6546891
rs113551605
rs114276102

i)

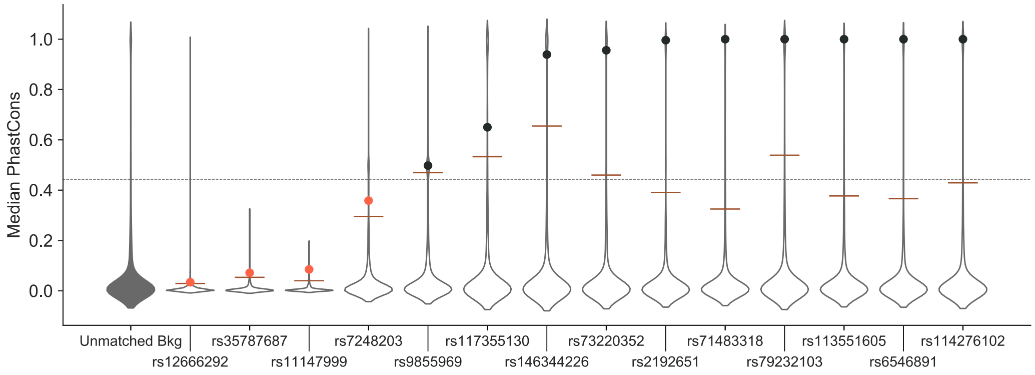
Median PhastCons
1.0
0.8
0.6
0.4
0.2
0.0
Unmatched Bkg
rs12666292
rs35787687
rs11147999
rs7248203
rs9855969
rs117355130
rs146344226
rs73220352
rs2192651
rs71483318
rs79232103
rs113551605
rs6546891
rs114276102

j)

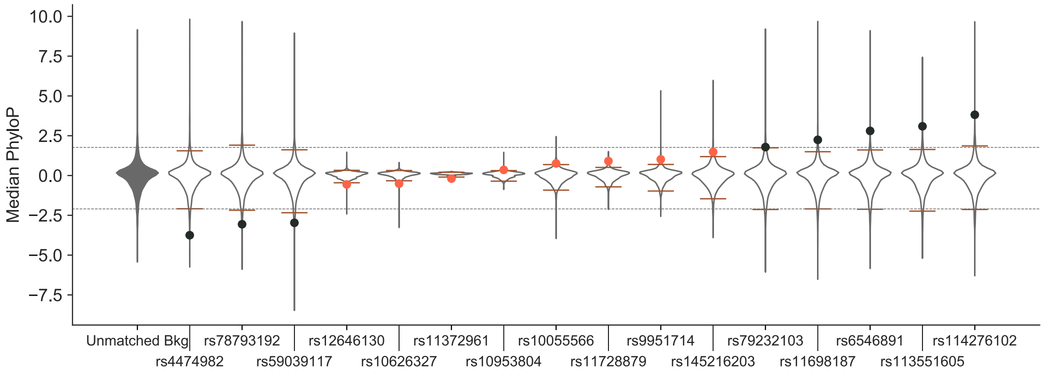
Median PhyloP
10.0
7.5
5.0
2.5
0.0
−2.5
−5.0
−7.5
Unmatched Bkg
rs4474982
rs78793192
rs59039117
rs12646130
rs10626327
rs11372961
rs10953804
rs10055566
rs11728879
rs9951714
rs145216203
rs79232103
rs11698187
rs6546891
rs113551605
rs114276102

k)

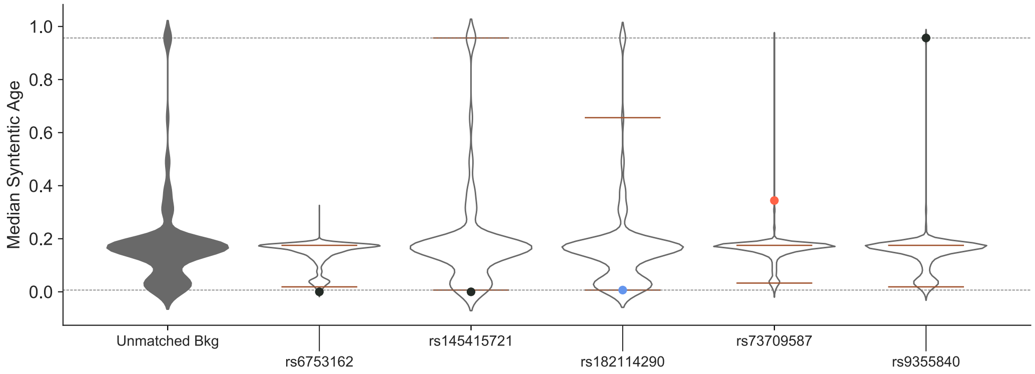
Median Syntentic Age
1.0
0.8
0.6
0.4
0.2
0.0
Unmatched Bkg
rs6753162
rs145415721
rs182114290
rs73709587
rs9355840

l)

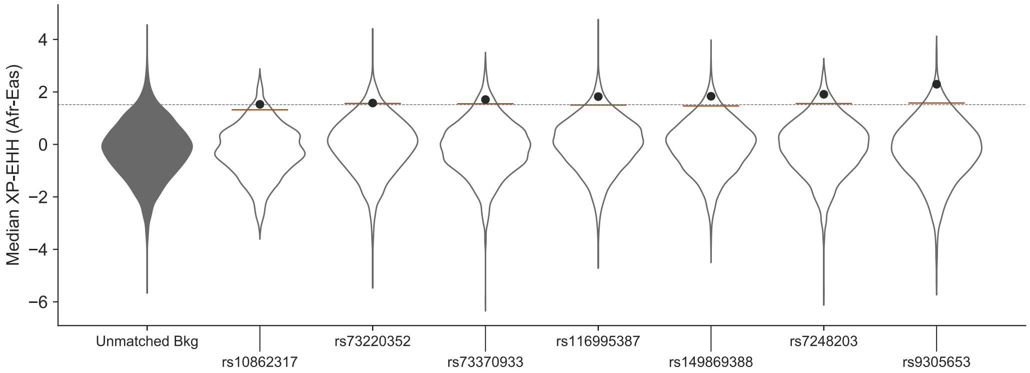
Median XP-EHH (Afr-Eas)
4
2
0
−2
−4
−6
Unmatched Bkg
rs10862317
rs73220352
rs73370933
rs116995387
rs149869388
rs7248203
rs9305653

m)

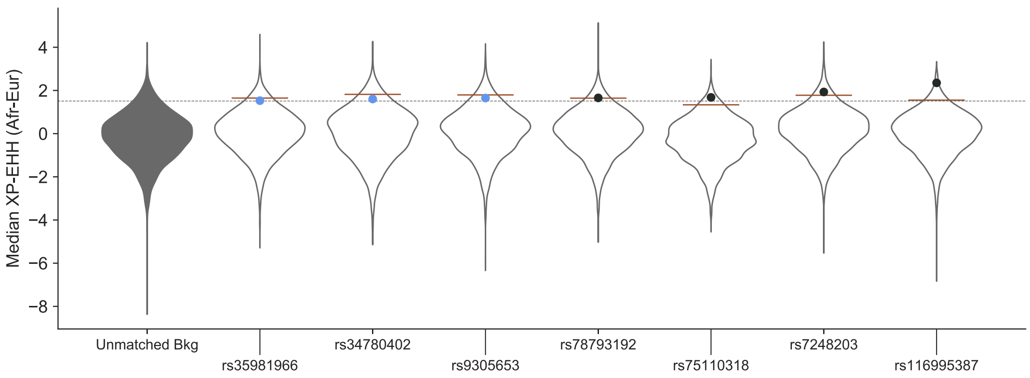
Median XP-EHH (Afr-Eur)
4
2
0
-2
-4
-6
-8
Unmatched Bkg
rs35981966
rs34780402
rs9305653
rs78793192
rs75110318
rs7248203
rs116995387

n)

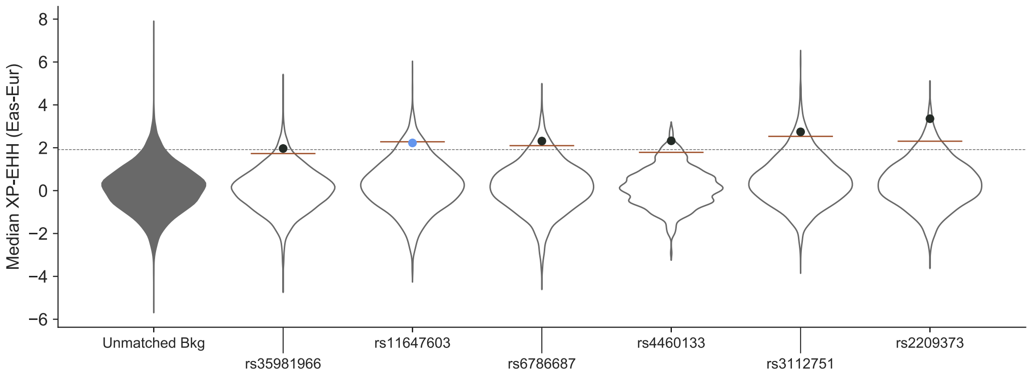
Median XP-EHH (Eas-Eur)
8
6
4
2
0
-2
-4
-6
Unmatched Bkg
rs35981966
rs11647603
rs6786687
rs4460133
rs3112751
rs2209373

Supplementary Figure 3: Sensitivity analysis of diverse evolutionary forces acting on sPTB-associated genomic regions

We tested 215 sPTB-associated genomic regions tested for diverse evolutionary forces (as described in Figure 2 and Methods). Heatmaps show sPTB-associated genomic region (columns) referred to by the lead SNP rsID and evolutionary forces (y-axis). The relative strength (size of square) and direction (color) of each selection measure for each PTB region is presented as a z-score calculated from that region's background control distribution. Statistical significance was assessed by comparing the median value of the evolutionary measure to the background control distribution to derive an empirical one or two-sided p-value (*p<0.05). Allele age (TMRCA from ARGweaver), PhyloP, and alignment block age are bi-directional measures, therefore, we calculated two-tailed p-values; all other evolutionary measures used one-tailed p-values. We evaluated the pattern of evolutionary forces observed under three different p-value thresholds for the lead variant sPTB GWAS p-value in each sPTB-associated genomic region: a) p<10E-4, b) 5*10E-5, c) 1*10E-5. Across all these thresholds we observed strong signals of diverse evolutionary forces.

a)

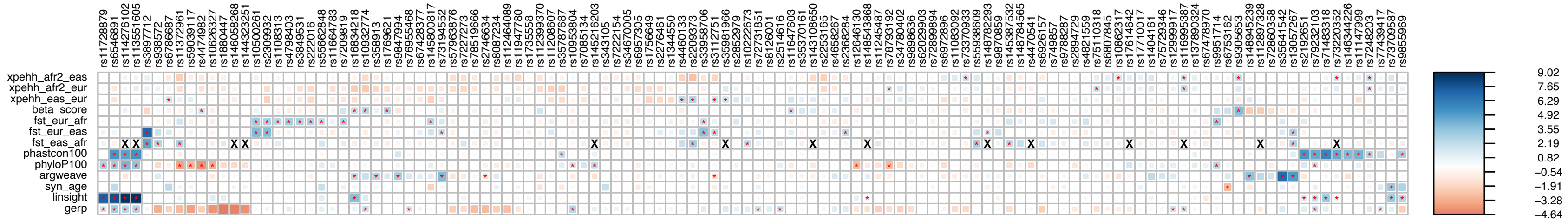

b)

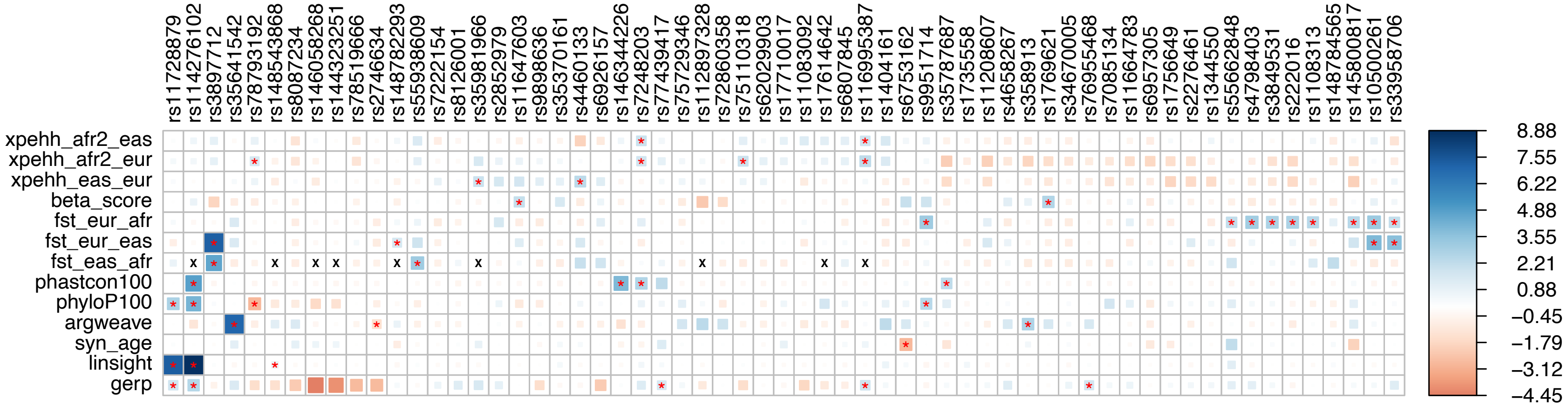
rs11728879
rs114276102
rs3897712
rs35641542
rs78793192
rs148543868
rs8087234
rs146058268
rs144323251
rs78519666
rs2746634
rs148782293
rs55938609
rs7222154
rs8126001
rs35981966
rs2852979
rs11647603
rs9898636
rs35370161
rs4460133
rs6926157
rs146344226
rs7248203
rs77439417
rs75729346
rs112897328
rs72860358
rs75110318
rs62029903
rs17710017
rs11083092
rs17614642
rs6807845
rs116995387
rs1404161
rs6753162
rs9951714
rs35787687
rs1735558
rs11208607
rs4658267
rs358913
rs1769621
rs34670005
rs76955468
rs7085134
rs11664783
rs6957305
rs1756649
rs2276461
rs1344550
rs55662848
rs4798403
rs3849531
rs222016
rs1108313
rs148784565
rs145800817
rs10500261
rs33958706
xpehh_afr2_eas
xpehh_afr2_eur
xpehh_eas_eur
beta_score
fst_eur_afr
fst_eur_eas
fst_eas_afr
phastcon100
phyloP100
argweave
syn_age
linsight
gerp
8.88
7.55
6.22
4.88
3.55
2.21
0.88
−0.45
−1.79
−3.12
−4.45

c)

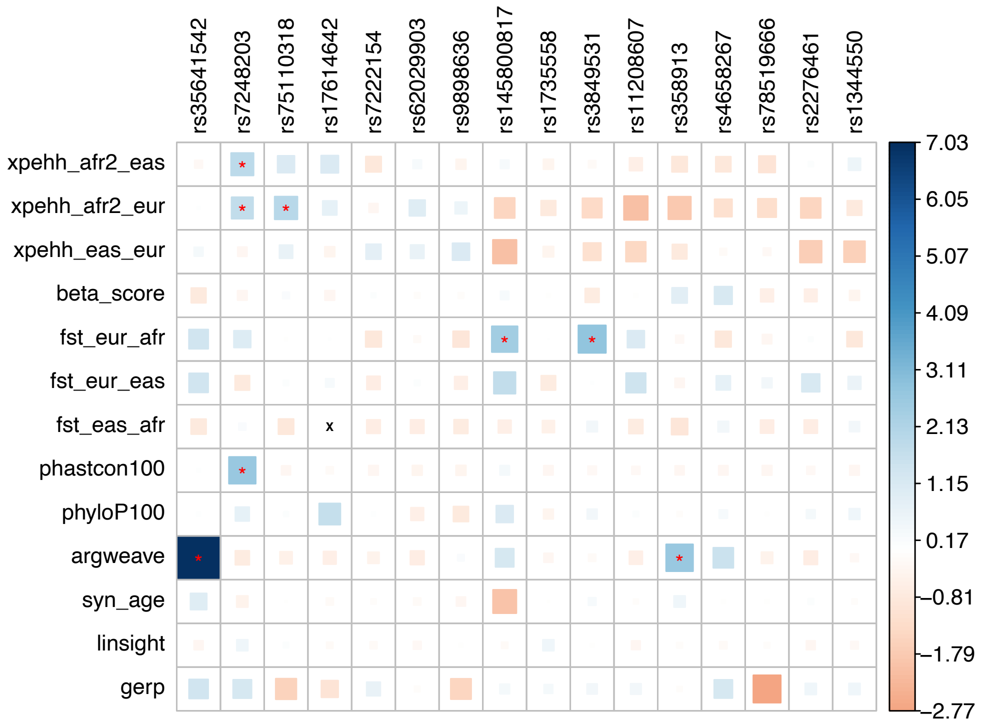
rs35641542
rs7248203
rs75110318
rs17614642
rs7222154
rs62029903
rs9898636
rs145800817
rs1735558
rs3849531
rs11208607
rs358913
rs4658267
rs78519666
rs2276461
rs1344550
xpehh_afr2_eas
xpehh_afr2_eur
xpehh_eas_eur
beta_score
fst_eur_afr
fst_eur_eas
fst_eas_afr
phastcon100
phyloP100
argweave
syn_age
linsight
gerp
7.03
6.05
5.07
4.09
3.11
2.13
1.15
0.17
−0.81
−1.79
−2.77
