# Supplementary material for: Accounting for diverse evolutionary forces reveals mosaic patterns of selection on human preterm birth loci
Source: Nat Commun. 2020 Jul 24;11:3731. doi: 10.1038/s41467-020-17258-6 (PMC7382462; doi:10.1038/s41467-020-17258-6)
Supplement: Supplementary file 3 — Reporting Summary [file 41467_2020_17258_MOESM3_ESM.pdf]

nature research

Corresponding author(s): Antonis Rokas & John A. Capra

Last updated by author(s): 2020-6-15

Reporting Summary

Nature Research wishes to improve the reproducibility of the work that we publish. This form provides structure for consistency and transparency in reporting. For further information on Nature Research policies, see our Editorial Policies and the Editorial Policy Checklist.

Statistics

For all statistical analyses, confirm that the following items are present in the figure legend, table legend, main text, or Methods section.

n/a	Confirmed	
☐	☒	The exact sample size (n) for each experimental group/condition, given as a discrete number and unit of measurement
☐	☒	A statement on whether measurements were taken from distinct samples or whether the same sample was measured repeatedly
☐	☒	The statistical test(s) used AND whether they are one- or two-sided<br>*Only common tests should be described solely by name; describe more complex techniques in the Methods section.*
☒	☐	A description of all covariates tested
☐	☒	A description of any assumptions or corrections, such as tests of normality and adjustment for multiple comparisons
☐	☒	A full description of the statistical parameters including central tendency (e.g. means) or other basic estimates (e.g. regression coefficient) AND variation (e.g. standard deviation) or associated estimates of uncertainty (e.g. confidence intervals)
☐	☒	For null hypothesis testing, the test statistic (e.g. F, t, r) with confidence intervals, effect sizes, degrees of freedom and P value noted<br>*Give P values as exact values whenever suitable.*
☒	☐	For Bayesian analysis, information on the choice of priors and Markov chain Monte Carlo settings
☒	☐	For hierarchical and complex designs, identification of the appropriate level for tests and full reporting of outcomes
☒	☐	Estimates of effect sizes (e.g. Cohen's d, Pearson's r), indicating how they were calculated

Our web collection on statistics for biologists contains articles on many of the points above.

Software and code

Policy information about availability of computer code

Data collection	Weir and Cockerham's FST calculated with VCFTools (v0.1.14, https://vcftools.github.io/index.html)<br>Haplotype metrics (his, iES, xp-EHH) calculated with rehh (v2.0, https://cran.r-project.org/web/packages/rehh/index.html)<br>Beta Score calculated with BetaScan (beta-1 version; https://github.com/ksiewert/BetaScan)<br>Alignment Block Age calculated from 100-way species alignment obtained from UCSC (http://hgdownload.cse.ucsc.edu/goldenpath/hg19/multiz100way/)<br>Binary haplotypes using the IMPUTE function of vcftools (v0.1.15.)<br>PHAST package (https://github.com/CshlSiepelLab/phast)<br>biomaRt package (https://bioconductor.org/packages/release/bioc/html/biomaRt.html)<br>GTEx_edit.pl – Script extract the relevant data from the GTEx files<br>haploreg_edit.pl - Script to extract the important information from filtered haploreg data<br>vep_edit.pl – Script to extract and edit VEP data<br>biomart.R – Script to retrieve hg18, hg19 and hg38 positions based on rs number
Data analysis	Median joining networks were created using PopART (http://popart.otago.ac.nz/index.shtml)<br>Variant clumping & haplotypes with PLINK1.9b3s (pngu.mgh.harvard.edu/purcell/plink/)<br>ape - https://cran.r-project.org/web/packages/ape/index.html<br>phangorn - https://cran.r-project.org/web/packages/phangorn/index.html<br>phytools - https://cran.r-project.org/web/packages/phytools/index.html<br>geiger - https://cran.r-project.org/web/packages/geiger/index.html<br>readr - https://cran.r-project.org/web/packages/readr/index.html<br>VCFtools https://vcftools.github.io/index.html<br>split_multiz_v2.pl – combine variant data from multiple sources and produce XMFA

xmfa_parsimony.pl – Calculates ancestral reconstruction for each variant
calc_r2.py - Calculates pairwise ld for input gwas snps using plink.
calc_summary_stat.py - Calculates z-scores for each input locus per annotation across control snps.
clump_snps.py - Takes GWAS summary statistics file 1) run plink clump and 2) bin all gwas variants by different levels of LD.
combine_annotations.py - Combines many output files from extract_from_bed.py
combine_control_sets.pl - Combines all the control snps with ld snps into one text file. One column per control set.
expand_control_set.py - Takes gwas clumped input snps and its corresponding SNPSNAP control snps and add control snps in ld with lead snps.
extract_all_snps_from_bed.py - Takes all control snps, split by chrm, run bedtools, and intersect on annotation file.
extract_from_bed.py - Runs bedtools intersect on control set snps against a given annotation file.
get_ld_partners.py - Calculates LD partners.
get_rsID_from_input_gwas.py - Gets rsID from the input GWAS for given chr:pos coordinates.
plot_annotation.py - Creates violin plots comparing the gwas input loci value for an annotation compared to control sets and will also calculate an empirical p-value per loci
python_slurm_script.slurm Runs clump_snps.py
qc_sets.py - Run after running expand_control_set.py. Searches through all files in log\, matching_sets\, and *_annotate\, then extracts the min and max value of the control sets from the file names, and reports missing sets in between min and max value
snpsnap_overlap_with_annotation.py - Run bedtools intersect on snpsnap database snps and user specified annotation files.
summarize_sets.py - Run after running expand_control_set.py. Concatenates the matching_summary.tsv file for each control set into one file and then summarize by various quality metrics and create plots
updated_control_sets.py - Run after combine_control_sets.py. Adds columns to the combine_control_sets.tsv file if that control set does not exist.

All custom data scripts can be found on FigShare DOI: 10.6084/m9.figshare.c.4602905.

For manuscripts utilizing custom algorithms or software that are central to the research but not yet described in published literature, software must be made available to editors and reviewers. We strongly encourage code deposition in a community repository (e.g. GitHub). See the Nature Research guidelines for submitting code & software for further information.

Data

Policy information about availability of data

All manuscripts must include a data availability statement. This statement should provide the following information, where applicable:

- Accession codes, unique identifiers, or web links for publicly available datasets
- A list of figures that have associated raw data
- A description of any restrictions on data availability

All the data used in this study were obtained from the public domain (see the URLs below) or deposited in a figshare repository at DOI: 10.6084/m9.figshare.c.4602905.

Publicly available data was downloaded from the following sources. PhyloP, PhastCons, 100-way species alignment and GERP data was obtained from the UCSC genome browser (http://hgdownload.cse.ucsc.edu/goldenPath/hg19/phyloP100way/ , http://hgdownload.cse.ucsc.edu/goldenPath/hg19/phastCons100way/ , http://hgdownload.cse.ucsc.edu/goldenpath/hg19/multiz100way/ , and http://genome.ucsc.edu/cgi-bin/hgTrackUi?db=hg19&g=allHg19RS_BW . LINSIGHT data was obtained from https://github.com/CshlSiepelLab/LINSIGHT. Thousand genomes phase 3 data was obtained from http://www.internationalgenome.org/ . TMRCA from ARGWEAVER was obtained from http://compgen.cshl.edu/ARGweaver/CG_results/download/ .

Field-specific reporting

Please select the one below that is the best fit for your research. If you are not sure, read the appropriate sections before making your selection.

[x] Life sciences   [ ] Behavioural & social sciences   [ ] Ecological, evolutionary & environmental sciences

For a reference copy of the document with all sections, see nature.com/documents/nr-reporting-summary-flat.pdf

Life sciences study design

All studies must disclose on these points even when the disclosure is negative.

Sample size	The data used in this study was the 10,000 variants with the strongest associated with spontaneous preterm-birth generated by Zhang et. al. 2017. We selected variants associated with spontaneous preterm-birth based on the PTB GWAS p-value. The top 10,000 variants were inclusive of all sPTB associated variants that met the GWAS p-value threshold (10E-4).
Data exclusions	The original dataset of 10,000 variants was filtered for lead-variants with a p-value of 10E-4 before any downstream analysis. This was done to filter out the variants least likely associated with sPTB.
Replication	Biological or technical replicates do not apply to our computational analysis of sPTB associated variants.
Randomization	NA
Blinding	Blinding was not applicable for this analysis since selection of sPTB associated variants were selected based on GWAS P-value before any downstream analysis. No treatment of interventions are used in this study. Results reported are based on an unbiased quantitation based on

randomly selected background distributions.

Reporting for specific materials, systems and methods

We require information from authors about some types of materials, experimental systems and methods used in many studies. Here, indicate whether each material, system or method listed is relevant to your study. If you are not sure if a list item applies to your research, read the appropriate section before selecting a response.

Materials & experimental systems

n/a	Involved in the study
☒	☐ Antibodies
☒	☐ Eukaryotic cell lines
☒	☐ Palaeontology and archaeology
☒	☐ Animals and other organisms
☒	☐ Human research participants
☒	☐ Clinical data
☒	☐ Dual use research of concern

Methods

n/a	Involved in the study
☒	☐ ChIP-seq
☒	☐ Flow cytometry
☒	☐ MRI-based neuroimaging
