# Supplementary material for: Accounting for diverse evolutionary forces reveals mosaic patterns of selection on human preterm birth loci
Source: Nat Commun. 2020 Jul 24;11:3731. doi: 10.1038/s41467-020-17258-6 (PMC7382462; doi:10.1038/s41467-020-17258-6)
Supplement: Supplementary file 4 — Description of Additional Supplementary Files [file 41467_2020_17258_MOESM4_ESM.pdf]

Description of Additional Supplementary Files

File Name: Supplementary Data 1
Description: **sPTB lead SNPs and LD SNPs for independent sPTB genomic regions.** Independent sPTB regions denoted by their lead SNP (SNP with lowest P-value) and SNPs in LD after clumping Zhang et. al. sPTB GWAS.

File Name: Supplementary Data 2
Description: **Heatmap z-score results in tabular format for Figure 2.** Each row represents an independent sPTB associated region denoted by the lead SNP (column 'rsid') and corresponding zscore for evolutionary measures (columns).

File Name: Supplementary Data 3
Description: **Heatmap p-value results in tabular format for Figure 2.** Each row represents an independent sPTB associated region denoted by the lead SNP (column 'rsid') and the corresponding empirical p-value obtained by comparing to the matched background distribution for difference evolutionary measures (columns).

File Name: Supplementary Data 4
Description: **1KG Individual IDs used for analysis**. Individual IDs and corresponding 1000 Genomes superpopulation assignment used for calculating evolutionary measures.

File Name: Supplementary Data 5
Description: **Cluster assignment and functional annotation for sPTB genomic regions.** The included regions are those with -1.5 ≥ z-score ≥ 1.5 from Figure 2. Each row represents the lead SNP and SNPs in LD for sPTB associated regions with an absolute value z-score ≥ 1.5 for any of the evolutionary measures evaluated. Columns include corresponding data for genomic position, nearest gene, statistics fro
